# PUS1 is a novel biomarker for evaluating malignancy of human renal cell carcinoma

**DOI:** 10.18632/aging.204799

**Published:** 2023-06-13

**Authors:** Lin Li, Chongying Zhu, Shouying Xu, Qiang Xu, Da Xu, Sishun Gan, Xingang Cui, Chao Tang

**Affiliations:** 1 National Clinical Research Center for Child Health of the Children’s Hospital, Zhejiang University School of Medicine, Hangzhou 310052, China; 2 Department of Urology, Third Affiliated Hospital of the Second Military Medical University, Shanghai 201805, China; 3 Department of Obstetrics and Gynecology, Ruijin Hospital, Shanghai Jiaotong University School of Medicine, Shanghai 200025, China; 4 Department of Urology, Xinhua Hospital, School of Medicine, Shanghai Jiaotong University, Shanghai 200092, China

**Keywords:** PUS1, renal cell carcinoma, biomarker, malignancy

## Abstract

Renal cell carcinoma (RCC) is one of the most common malignancies. Despite the rapid development of the oncology research and surgical treatment, the prognosis of RCC has not significantly improved. Thus, exploration of the pathological molecular mechanism and development of new therapeutic targets of RCC are of great importance. Herein, by bioinformatic analysis and *in vitro* cell experiments, we report that, the expression of pseudouridine synthase 1 (PUS1), belonging to the family of PUS enzymes that participate in RNA modifications, is closely associated with RCC progression. In addition, the upregulated PUS1 expression results in the elevated RCC cancer cell viability, migration, invasion and colony formation ability, whereas the decreased PUS1 expression exerts the opposite effects on RCC cells. Thus, our findings show the potential role of PUS1 in RCC cells, providing with evidence that PUS1 is involved in RCC progression, which may help contribute to RCC diagnosis and intervention in clinic.

## INTRODUCTION

As shown by the latest data of cancer from the United States, renal cell carcinoma (RCC), which is recognized as the most common malignancy of the genitourinary system, ranks the sixth most common cancer in men, with 65000 new cases and 15000 deaths per year [1]. Despite the rapid development of the research in cancer and clinical surgical treatment, the prognosis of RCC has not significantly improved in clinic at present. Due to the shortage of effective molecular bio-markers for the RCC treatment, plenty of patients with renal cancer consequently develop to advanced stage [2, 3]. In recent years, although targeted agents have been applied, showing the function in prolonging the survival and prognosis particularly in patients with tumor metastases, the median survival of patients remains less than three years [4]. Moreover, drug resistance and economic burden have become the two major problems in clinical practice [5]. Thus, exploration of the pathological molecular mechanism and development of promising therapeutic targets for RCC are of great importance for better treatment of RCC.

Accumulating evidence has suggested that RNA modifications play a pivotal role in epigenetic regulation of gene expression in sustaining physiological functions, and dysregulation of RNA modifications gives rise to various diseases, including cancer [6–8]. As an isomer of uridine, pseudouridine is found to be the most abundant RNA modification and is recognized to be the fifth RNA nucleotide [9, 10]. Previous studies have demonstrated that the isomerization process of uridine to pseudouridine can be triggered by either of the two following mechanisms: (1) by a small nucleolar RNA-dependent mechanism where the box H/ACA ribonucleoproteins are incorporated; (2) by an RNA-independent mechanism that requires the stand-alone pseudouridine synthase (PUS) enzymes [11, 12]. To date, the PUS enzymes can be divided into six families, including the TruA, TruB, TruD, RsuA, RluA and Pus10 families [13]. While RNA pseudouridylation has been suggested to be associated with human physiological functions and diseases [14, 15], the biological regulation of PUS remains largely unknown.

As an important member belonging to the pseudouridine synthase family, PUS1 is found to participate in regulating the pseudouridylation modification of almost all kinds of RNAs, including tRNAs, ncRNAs, mRNAs, snoRNAs, and snRNAs [16, 17]. Despite its role in various diseases such as myopathy, lactic acidosis and sideroblastic anaemia (MLASA) syndrome [18, 19] where *PUS1* gene is mutated, recent studies additionally elucidated the versatile role of PUS1 in cancer. In a latest report, PUS1 expression was shown to be positively correlated with triple-negative breast cancer (TNBC) status and tumor grade, and thereby can be potentially applied for predicting poor outcomes and triple-negative status in breast cancer [16].

In this study, by bioinformatic analysis, experiments *in vitro* and clinical RCC samples, we report herein that PUS1 expression was closely associated with RCC progression and the upregulated PUS1 expression results in the elevated RCC cancer cell viability and mobility, providing with the evidence that PUS might be a promising treatment target for RCC therapy.

## MATERIALS AND METHODS

### Data collection

The gene expression, mRNA sequencing (mRNA) expression and clinical information of 532 kidney renal clear cell carcinoma samples were retrieved from the Cancer Genome Atlas (TCGA) database (https://portal.gdc.cancer.gov/). The normal kidney composed of 72 TCGA samples and 89 GTEx database. GTEx datasets are available at the GTEx data portal (https://www.gtexportal.org/home/datasets) for the current release (V8). The four GEO clear cell renal cancer cohorts (GSE15641, GSE17818, GSE17895, and GSE40435) were obtained from the Gene-Expression Omnibus (GEO) (https://www.ncbi.nlm.nih.gov/geo/) as MINiML files. In GSE15641, 23 control samples and 32 clear cell renal samples were enrolled; in GSE17818, 13 normal patients and 103 renal clear cell carcinoma patients; in GSE17895, the data contained 22 health renal samples and 138 patients; and the GSE40435 dataset had included 101 health kidney and 101 kidney clear cell carcinoma.

### Biological data processing

For 14 TCGA cancer types with over ten paired tumor and normal samples (THCA, KIRP, BLCA, LIHC, HNSC, BRCA, LUAD, PRAD, ESCA, KICH, LUSC, KIRC, STAD, COAD), we performed GSCA differential expression analysis using normalized and batch corrected RSEM mRNA expression. We calculated the fold change by dividing the mean of the tumor by the mean of the normal, and our *p*-value is estimated by a *t*-test and then adjusted by the Fisher’s exact test. The data from GEO contain the platform, samples and GSE records. The microarray data were normalized by the R (version 3.4.1) preprocess core package named “Normalize. Quantiles” following the log_2_ transformation. Gene symbols were generated from probes based on the annotation information in the normalized data. The Kaplan-Meier (KM) survival analysis with log-rank test was applied for comparing the survival difference between the above two groups. For KM curves, *p*-values and hazard ratio (HR) with 95% confidence interval (CI) were generated by log-rank tests and univariate Cox proportional hazards regression as described previously [20]. All analytical methods above and R packages were performed using R software. *P* < 0.05 was considered as statistically significant.

### Enrichment analysis

After using limma package to study the differential expression of the mRNA from TCGA, a functional enrichment analysis was conducted to further confirm the underlying function of potential targets. The Gene Ontology (GO) analysis and Kyoto Encyclopedia of Genes and Genomes (KEGG) analysis were performed to annotate genes with functions. The ClusterProfiler package in R was used to analyze the GO function and enrich the KEGG pathway. The R software ggplot2 package was employed to draw boxplot, and the pheatmap package was employed to draw heatmap.

### Construction and assessment of the nomogram

We further conducted Univariate and multivariate cox regression analysis to identify the proper terms to build the nomogram as described previously [21]. The forest showed the *P* value, HR and 95% CI of PUS1, age, gender, TNM stage and grade by “forestplot” R package. Multivariate cox proportional hazards analysis was used to develop a nomogram to predict 1-year, 3-year and 5-year overall recurrence. Based on the nomogram’s points associated with each risk factor, an individual patient’s risk of recurrence can be calculated by “RMS” R package.

### The renal cancer cell line analysis

The mRNA expression matrix of 33 renal cancer cell lines was downloaded from the Cancer Cell Line Encyclopedia (CCLE) portal (https://www.broadinstitute.org/ccle) and the analysis was generated by the R ggplot2 package.

### Cell culture

Human embryonic kidney 293T (HEK293T) cells and human RCC cell lines, including Caki-1, 786-O, ACHN, OSRC2, A498, Caki-2 and 769P cells, were purchased from the Chinese Academy of Sciences (Shanghai, China). HEK293T cells were maintained in high glucose DMEM (Hyclone, Logan, UT, USA) supplemented with 10% (v/v) fetal bovine serum (FBS, Life Technologies, Inc., Grand Island, NY). ACHN cells were cultured in MEM medium (Hyclone, USA) supplemented with 10% FBS, and Caki-1, Caki-2, 786-O, OSRC2, A498 and 769P cells were all incubated in RPMI-1640 medium (Hyclone, USA) containing 10% FBS as described previously [22]. All the cells were grown as a monolayer on plastic cell culture dishes at 37°C in 5% CO_2_.

### Antibodies

Anti-PUS1 (#db3219) antibody was from DiagBio (Hangzhou, China) and HA-tag (AF2305) antibody was obtained from Beyotime Biotechnology (Shanghai, China). Glyceraldehyde-3-phosphate dehydrogenase (GAPDH, sc-32233) antibody was purchased from Santa Cruz Biotechnology (Santa Cruz, CA, USA).

### Plasmids, viruses and infections

The PUS1 lentiviral expression vector, pLV3-CMV-PUS1-3×HA-CopGFP-Puro, was constructed by Mr. Lin Li, and the pLV3-CMV-3×HA-CopGFP-Puro empty vector was used as a control. The lentiviral shRNA-expressing vector, pLKO.1-EGFP-PUS1-shRNA-puro, was constructed by Dr. Shouying Xu, and the pLKO.1-EGFP-puro empty vector carrying a scrambled sequence was used as a control. Lentiviruses were generated by co-transfecting the HEK293T packaging cells with lentiviral plasmids and packaging plasmids (PsPAX and PMD2.0G) as described previously [23]. The lentiviruses-containing supernatants with the titers greater than 1 × 10^6^ cfu/ml were used for subsequent infection in the presence of 8 μg/ml polybrene (Sigma-Aldrich, St. Louis, MO, USA). After infection, cells were selected with puromycin at a dose of 2 μg/mL for seven days, and then the cells were cultured with puromycin at a low dose and were subsequently prepared for following experiments.

### Western blotting

Total protein from human RCC Caki-1, 786-O, ACHN, OSRC2, A498, Caki-2 and 769P cells were obtained using RIPA lysis buffer (Beyotime, Shanghai, China), and protein concentrations were determined by standard Bradford assay with a commercial kit (Beyotime, Shanghai, China), followed by western blotting as described previously [24–26]. Briefly, 50 μg of total protein was subjected to SDS-PAGE followed by a transfer onto polyvinylidene fluoride (PVDF) membranes (Millipore, Bedford, MA, USA). Then, membranes were incubated with different primary antibodies at 4°C overnight, and were further incubated with corresponding secondary antibodies (Beyotime, Shanghai, China) the next day. Immunosignals were developed by using the Enhanced Chemiluminescence System.

### Cell counting kit-8 (CCK-8) assays

CCK-8 assays were conducted as described previously [26, 27]. Briefly, cells were seeded into 96-well plates and were infected with PUS1-expressing lentiviruses, control lentiviruses, PUS1-shRNA-expressing lentiviruses, or lentiviruses with scrambled shRNA. After continued culture for 48 h, cells in each well were subjected to 20 μl CCK-8 solution (Beyotime, Shanghai, China) and were cultured for 2 h. Cell viability was subsequently examined by measuring the absorbance at 450 nm *via* a multi-mode plate reader (Bio-Tek Instruments, Hopkinton, MA, USA).

### Wound healing assays

Wound healing assays were performed as previously described [28]. Briefly, 4 × 10^5^ cells were cultured in six-well plates and were infected with PUS1-expressing lentiviruses, control lentiviruses, PUS1-shRNA-expressing lentiviruses, or lentiviruses with scrambled shRNA, and were cultured for 24 h. Then, the cells were subjected to serum starvation for 12 h. After that, cells were rinsed with medium and the confluent cell layer was scratched with a sterile tip to generate an artificial wound. After 12 h, migrated cells were observed by microscopy and the distance between the edges of the “wound” was consequently analyzed.

### Matrigel invasion assays

Cell invasive capacity was determined by using Matrigel (BD Biosciences, Franklin Lakes, NJ, USA) coated Transwell inserts (6.5 μm, Costar, Cambridge, MA, USA) containing polycarbonate filters with pores (8 μm) as detailed previously [28]. Briefly, a mixture of Matrigel and culture medium at the volume proportion of 1:2 at 50 μl was enclosed by each membrane in Transwell inserts, respectively. After infection and culture for 48 h, 2 × 10^5^ cells in a total volume of 200 μl of serum-free medium were added in the upper chamber, while 500 μl of culture medium with 10% FBS was loaded to the corresponding lower well. After culture for another 24 h, non-invaded cells were gently removed from the upper surface of the filter, and the cells that had moved through the filter and stayed at the bottom of the Transwell membrane were fixed in ice-cold methanol and were then stained with 0.2% crystal violet-PBS solution (m/v) for 10 min at room temperature. Numbers of the invasive cells from the bottom of the Transwell membrane in six randomly selected fields were counted in each experiment, and the invasive cells were consequently observed and counted using a light microscope.

### Colony formation assays

Colony formation assay was performed as previously described [29]. Briefly, cells were infected with PUS1-expressing lentiviruses, control lentiviruses, PUS1-shRNA-expressing lentiviruses, or lentiviruses with scrambled shRNA. At 24 h after infection, cells were seeded in 6-well plates at a density of 1000 cells/well in 2 ml culture medium. After growing for two weeks, colonies were stained with 0.5% crystal violet solution at room temperature, and colonies were subsequently counted, photographed and statistically analyzed.

### RCC tissue sample obtainment

A total of 10 pairs of RCC tissues and adjacent non-tumor tissues were collected from patients undergoing surgery at the Third Affiliated Hospital of the Second Military Medical University from 2021 to 2022. For inclusive criteria, patients didn’t receive any chemotherapy or radiotherapy before operation. All the tumor tissues and paired adjacent non-tumor tissues were identified by pathologists. A protocol associated with this study was approved by the Third Affiliated Hospital of the Second Military Medical University institutional review board, and informed consent was obtained from each patient.

### Statistical analysis

Data were expressed as mean ± SD, and were analyzed by one-way ANOVA and Tukey-Kramer multiple comparison test (SPSS 13.0J software; SPSS, Inc., Chicago, IL). *P* < 0.05 and *p* < 0.01 were considered with statistical significance.

### Availability of data and materials

All data generated or analyzed during this study are included in this published article.

### Consent for publication

The author declares that all work described here has not been published before and that its publication has been approved by all co-authors.

## RESULTS

### The expression of PUS1 is increased in renal cancer

**Figure 1:**
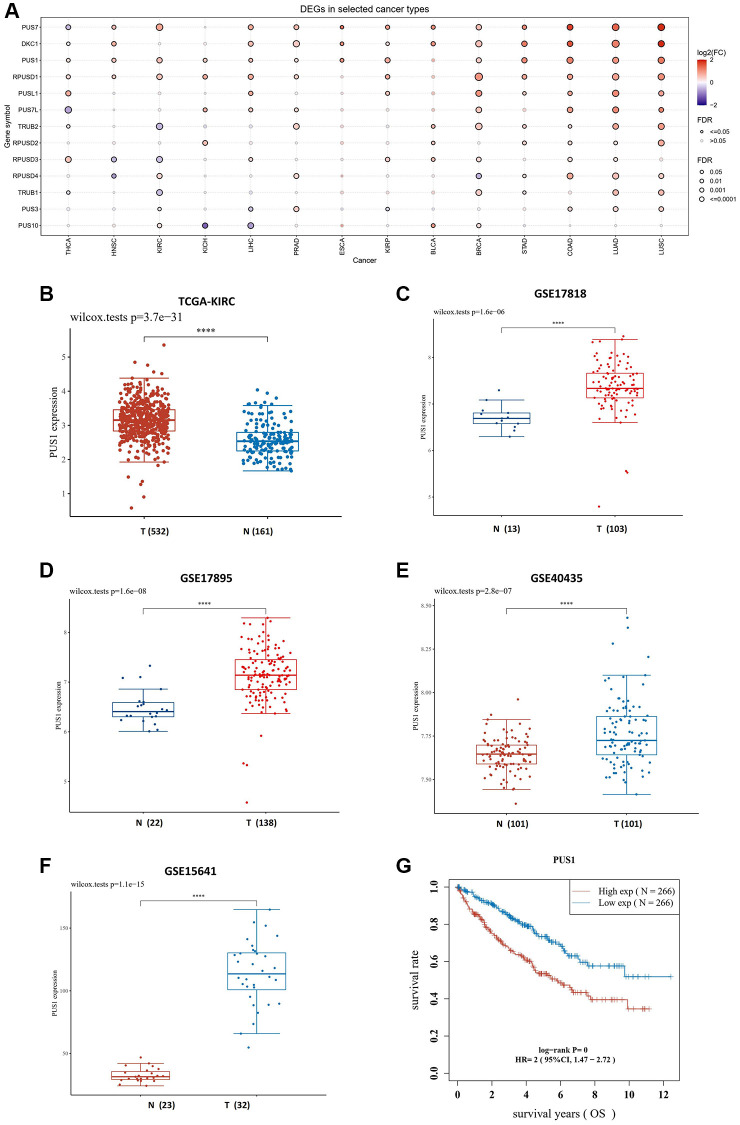
**The expression of PUS1 is increased in renal cancer.** (**A**) Expression of PUS family gene in pan-cancer. The color of bubbles represents the difference in expression of PUS family genes between tumor tissues and paired normal para-cancer tissues. Red: up-regulated expression in tumor tissues; blue, down-regulated expression in tumor tissue; bubble size indicates significant difference. (**B**) The expression of PUS1 in KIRC. Red: tumor tissue; blue: paired para-cancer normal tissue. (**C**–**F**) The PUS1 gene expression in four GEO datasets, including GSE17818 (**C**), GSE17895 (**D**), GSE40435 (**E**) and GSE15641 (**F**). (**G**) The overall survival (OS) of PUS1 in KIRC. Red: high expression group of PUS1; blue: low expression group of PUS1.

Firstly, we investigated the expression of pseudouridylate synthases family in pan-cancer using The Cancer Genome Atlas (TCGA) database online. Totally 14 TCGA cancer types were enrolled, including thyroid carcinoma (THCA), kidney renal papillary cell carcinoma (KIRP), bladder urothelial carcinoma (BLCA), liver hepatocellular carcinoma (LIHC), head and neck squamous cell carcinoma (HNSC), breast invasive carcinoma (BRCA), lung adenocarcinoma (LUAD), prostate adenocarcinoma (PRAD), esophageal carcinoma (ESCA), kidney chromophobe (KICH), lung squamous cell carcinoma (LUSC), kidney renal clear cell carcinoma (KIRC), stomach adenocarcinoma (STAD) and colon adenocarcinoma (COAD), which have over ten paired tumor and normal samples, correspondingly. According to our findings, the expression of *PUS1*, *PUS7*, *DKC1* and *RPUSD1* is highly expressed in most cancer types, and among those genes, the expression of *PUS1*, *PUS7* and *RPUSD1* is markedly upregulated in renal cancers, including KICH, KIRC and KIRP (Figure 1A, 1B). In addition, we also examined the *PUS1* gene expression in four Gene Expression Omnibus (GEO) datasets, including GSE15641, GSE17878, GSE17895 and GSE40435. As expected, the results exhibited the increase of *PUS1* expression in renal cancer (Figure 1C–1F). Consistently, the overall survival of KIRC between high levels of *PUS1* and low levels of *PUS1* revealed statistical significance (Figure 1G), indicating the higher expression of *PUS1* may be associated with prognosis of renal cancer.

### The differential mRNA expression between clear cell renal cancer and normal tissue

**Figure 2:**
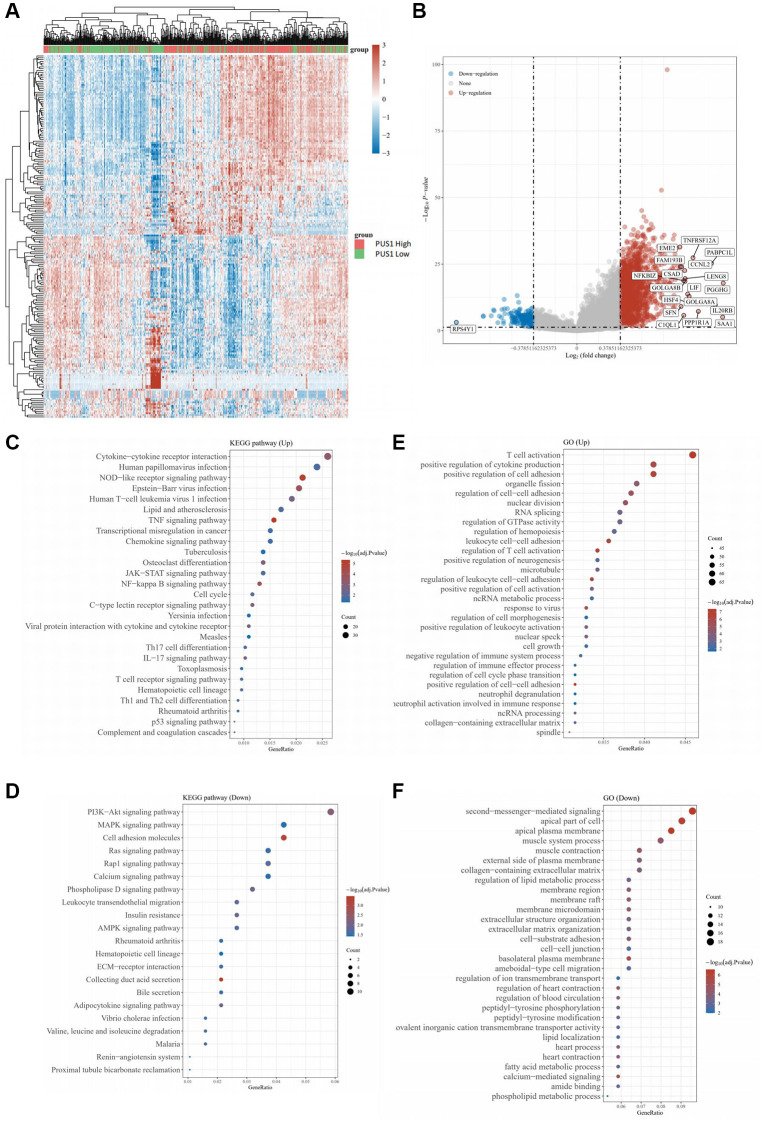
**The differential mRNA expression between clear cell renal cancer and normal tissue.** (**A**) Heatmap color indicates the differentially expressed genes (DEGs) by analyzing the KIRC data from TCGA database. (**B**) The volcano plots of DEGs in (**A**) were visualized. (**C**, **D**) GO analysis for enrichment of upregulated (**C**) and downregulated (**D**) signaling pathways significantly associated with PUS1 expression in KIRC. (**E**, **F**) KEGG analysis for enrichment of upregulated (**E**) and downregulated (**F**) signaling pathways significantly associated with PUS1 expression in KIRC.

We next analyzed the KIRC data from TCGA database and conducted the heatmap to compare the differentially expressed genes (DEGs) (Figure 2A). The volcano plots of DEGs were visualized between the cancer and normal tissues, and the value of log_2_(fold changes) above 1.2 or below −1.2 was defined as the threshold for the differential mRNA expression (Figure 2B). In total, 9708 DEGs were picked up ultimately, with 7324 up-regulated and 2384 down-regulated (table not shown). Noticeably, the results showed that the expression of genes, including *PUS1, PUS7L*, *RPUSD2*, *PUS10*, *RPUSD4*, *TRUB1*, and *DKC1*, was increased, while conversely, the expression of some genes was decreased, such as *PUSL1* (Figure 2B). To uncover the potential biological processes involved, we next performed the Gene Ontology (GO) analysis and obtained the enrichment for several cancer pathways of DEGs, which particularly focused on cell-cell adhesion and cytokine production, and intriguingly, results also suggested that pathways on immune related activity in T-cell activation were involved (Figure 2C, 2D). Similarly, we further conducted the Kyoto Encyclopedia of Genes and Genomes (KEGG) pathway enrichment analysis with the figure portraying several notable signal pathways, which included PD-1 checkpoint, PD-L1 expression and cell cycle (Figure 2E, 2F).

### Construction of a predictive nomogram

**Figure 3:**
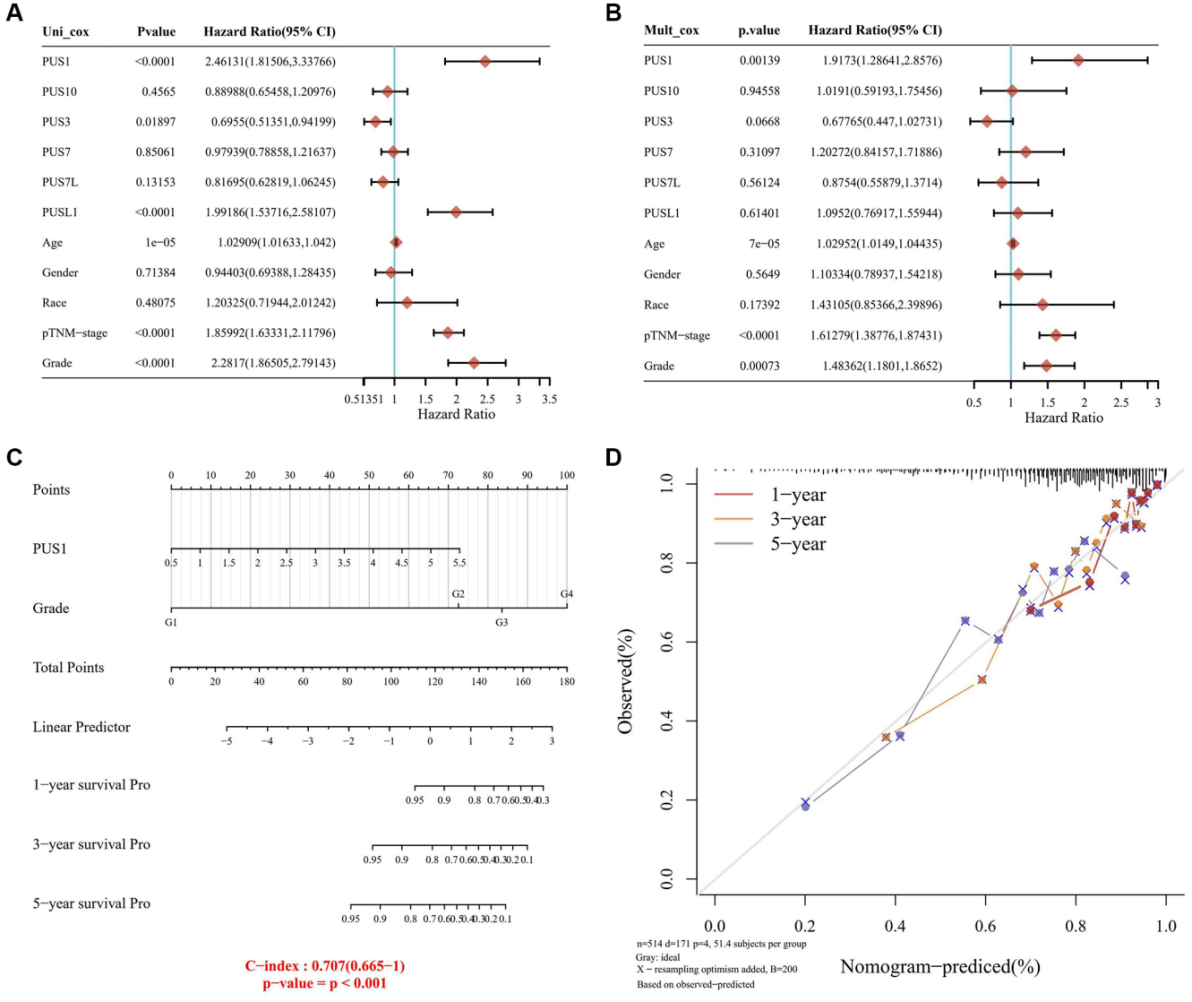
**Construction of a predictive nomogram.** (**A**, **B**) Univariate (**A**) and multivariate (**B**) Cox regression analyses were performed in renal cancer. (**C**) The nomogram gives 1-, 3- and 5-year overall survival in renal cancer. (**D**) The survival time of renal cancer dataset is displayed.

Considering a vital risk of *PUS1* for renal cancer patients, we further developed a clinically predictive model that was workable and may contribute to estimating the prognosis of renal cancer in clinic. We initially calculated the univariate and multivariate analysis of overall survival (OS) rate, including the expression of genes belonging to the PUS family (PUS1, PUS3, PUS7, PUS10, PUS7L, PUSL1), patients’ age, gender, race and Tumor Node Metastasis (TNM)-stage, and set grade as co-variate (Figure 3A, 3B). Intriguingly, our results provided a strong evidence that, similar as TNM-stage and tumor grade, PUS1 could be a potentially high risk for renal cancer, with a univariate cox regression of (HR 2.46131, 95% CI = 1.81506–3.33766, *P* < 0.0001) and a multivariate cox regression of (HR 1.9173, 95% CI = 1.28641–2.8576, *P* = 0.00139), which had already been accepted as predictive standards for renal cancer prognosis (Figure 3C). Next, we generated a nomogram for 1-year, 3-year, and 5-year renal clear cancer patients OS rate model in the discovery group. The elements of the model contained tumor grade and *PUS1* expression, with the nomogram showing a promising concordance with 1-year, 3-year, and 5-year OS (Figure 3D).

### Expression levels of PUS1 are associated with RCC cell malignancy

**Figure 4:**
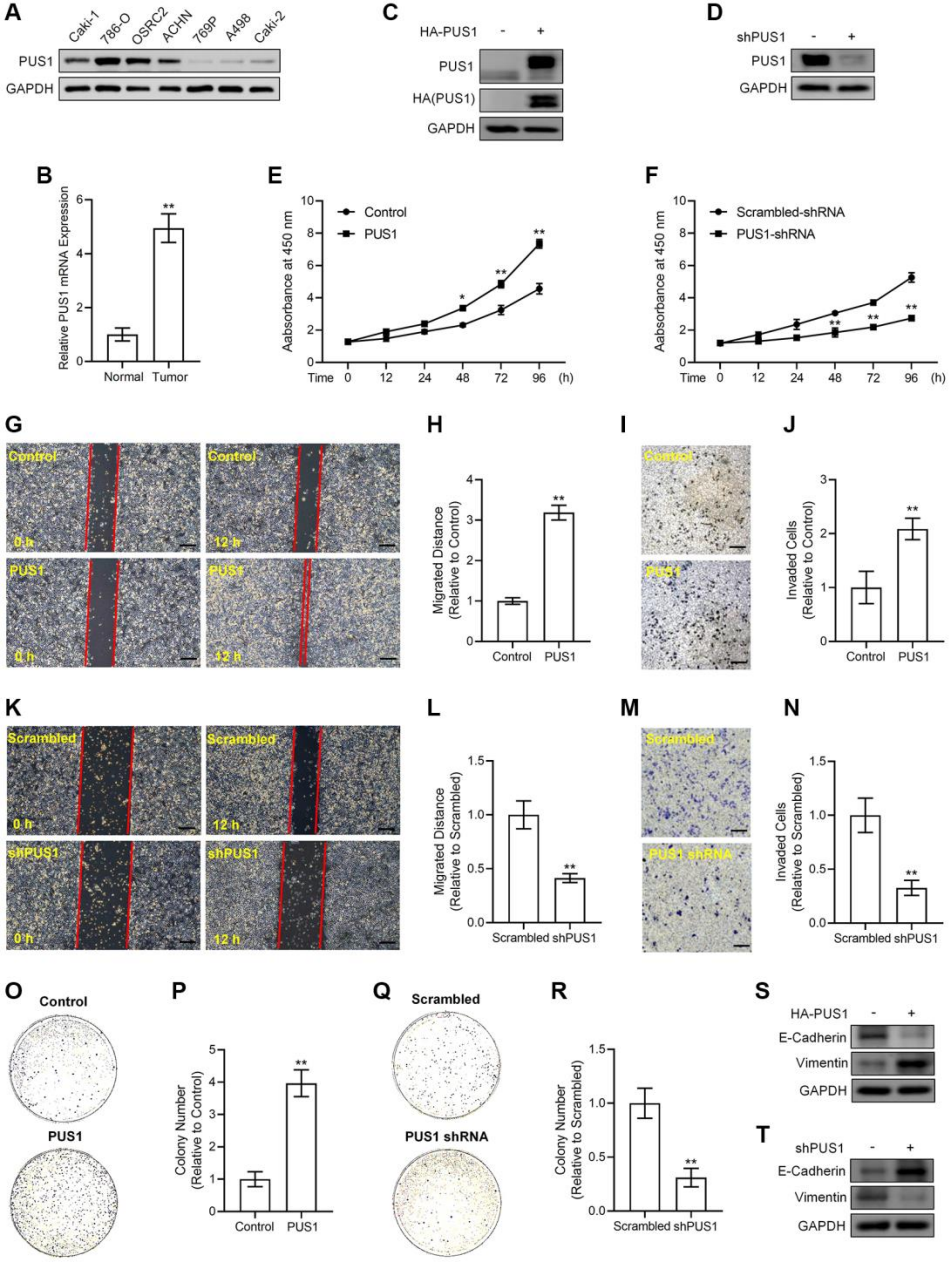
**Alterations in PUS1 expression affect A498 cell and ACHN cell viability, migration, invasion, colony formation, and the expression of E-Cadherin and Vimentin.** (**A**) The protein expression of PUS1 in different RCC cell lines. (**B**) The mRNA expression levels of *PUS1* in cancer tissues and adjacent normal epithelial tissues from patients with RCC. *N* = 10. (**C**) A498 cells are infected with PUS1-LV and PUS1 expression was detected by western blot. (**D**) ACHN cells are infected with PUS1-shRNA-LV and PUS1 expression was detected by western blot. (**E**) A498 cells are infected with PUS1-LV and cell viability was measured. (**F**) ACHN cells are infected with PUS1-shRNA-LV and cell viability was measured. (**G**) A498 cells are infected with PUS1-LV and cell migration was determined by wound healing assay. (**H**) Quantitative statistics of (**G**). (**I**) A498 cells are infected with PUS1-LV and cell invasion was detected by matrigel-transwell assay. (**J**) Quantitative statistics of (**I**). (**K**) ACHN cells are infected with PUS1-shRNA-LV and cell migration was determined by wound healing assay. (**L**) Quantitative statistics of (**K**). (**M**) ACHN cells are infected with PUS1-shRNA-LV and cell invasion was detected by matrigel-transwell assay. (**N**) Quantitative statistics of (**M**). (**O**) A498 cells are infected with PUS1-LV and cell colony formation was performed. (**P**) Quantitative statistics of (**O**). (**Q**) ACHN cells are infected with PUS1-shRNA-LV and cell colony formation was performed. (**R**) Quantitative statistics of (**Q**). (**S**) Protein levels of E-Cadherin and Vimentin in A498 cells infected with PUS1-LV. (**T**) Protein levels of E-Cadherin and Vimentin in ACHN cells infected with PUS1-shRNA-LV. SD, error bar; ^**^p < 0.01; ^*^p < 0.05.

To figure out whether dysregulation of PUS1 contributes to RCC progression, we examined the PUS1 protein expression in seven different RCC cell lines, including BCaki-1, 786-O, OSRC2, ACHN, 769P, A498 and Caki-2. Our results revealed that the expression of PUS1 in 786-O, OSRC2, ACHN cells was higher than other cell lines (Figure 4A), and the expression was particularly low in A498 cells (Figure 4A), which was consistent with the data of the renal cancer cell line analysis from the CCLE portal (Supplementary Figure 1). In addition, the mRNA expression of PUS1 was significantly increased in collected RCC tissues compared to that of paired normal tissues, reinforcing the notion that PUS1 may accelerate RCC progression (Figure 4B).

We next cloned human PUS1 coding DNA sequence (CDS) sequence and inserted it into the lentivirus vector with 3×HA tags to obtain the pLV3-CMV-PUS1-3×HA-CopGFP-Puro plasmid and generated 3×HA-PUS1 lentiviruses (PUS1-LV), whose expression in A498 cells was determined by western blot of detecting PUS1 and HA tag, respectively (Figure 4C). Meanwhile, we constructed the pLKO.1-EGFP-puro vector containing shRNA sequences targeting *PUS1* to inhibit endogenous PUS1 expression and generated *PUS1*-shRNA lentiviruses (PUS1-shRNA-LV) that significantly suppressed PUS1 protein expression in ACHN cells (Figure 4D). Infection of PUS1-LV significantly potentiated viability in a time dependent-manner, promoted migration, enhanced invasion, and increased colony numbers in A498 cells (Figure 4E, G–4J, 4O, 4P). Conversely, infection of PUS1-shRNA-LV resulted in the opposite effects in ACHN cells, including lowered viability at different time points, particularly at 48 h, 72 h and 96 h, down-regulated migration, suppressed invasive capability, and decreased colony numbers (Figure 4F, 4K–4N, 4Q, 4R). In consistence with the migration and invasion data, the expression of epithelial-mesenchymal transition (EMT)-related markers was altered simultaneously. Noticeably, overexpression of PUS1 by PUS1-LV decreased E-Cadherin protein expression but increased Vimentin protein levels in A498 cells (Figure 4S), whereas silencing of PUS1 by PUS1-shRNA-LV induced E-Cadherin expression but repressed Vimentin protein levels in ACHN cells contrarily (Figure 4T), suggesting PUS1 expression levels were associated with RCC cell mobility and metastasis. These results, which were in agreement with our bioinformatic analysis of the RNA sequencing data, prove that, PUS1 participates in RCC progression, indicating that PUS1 might have potential predictive value for prognosis of RCC in clinic.

## DISCUSSION

In our study, we found that RCC tissues showed the higher expression of PUS1 than that in normal tissues. As expected, bioinformatic analysis data revealed that the higher expression levels of PUS1 were correlated with higher tumor grade as well as poor prognosis of patients with RCC. Moreover, according to *in vitro* RNA sequencing analysis data, up-regulation of PUS1 expression affected a range of cancer related pathways. Consistently, by overexpression of PUS1 and knockdown of PUS1 in RCC cells *in vitro*, we showed that PUS1 expression was significantly associated with cancer cell malignancy, including cell viability, migration, invasion and colony formation ability.

RNA modifications play a vital role in regulating RNA functions and in controlling gene expression. As an important component of translation machinery, transfer RNA (tRNA) is processed and matured with various types of modifications [30]. Among which, pseudouridine is found to be the most abundant modification in RNA, which has been detected in almost all RNA species, but the biological functions of pseudouridine modification in human physiology and diseases remain largely unknown [31]. Previous studies have shown that urinary pseudouridine nucleoside levels are obviously higher in patients with cancer, providing with the possibility that urinary pseudouridine levels may potentially be used as a tumor bio-marker in clinic [32, 33]. Nevertheless, the clues for a direct function of pseudouridine modification and for the various PUS enzymes in carcinogenesis have not been obtained, despite a recent study indicating a potential role for PUS7 in myelodysplastic syndromes [15]. In the study presented here, we have demonstrated a direct role of PUS1 in RCC tumorigenesis. Particularly, we have proved that PUS1 regulates the growth and movement of RCC cells, providing with direct evidence for PUS in modulating urinary tumorigenesis.

Given that high expression of PUS1 is correlated with poor prognosis in RCC, we therefore hypothesized that *PUS1* could be an oncogene in RCC. As expected, both KEGG pathway analysis and GO bioinformatic analysis indicate that the altered expression of PUS1 is related to multiple pathways, including cancer pathways and cell function progressions, such as cell proliferation, cell growth, cell movement, cell death and cell differentiation. Consistently, our data of *in vitro* experiments in RCC cell lines demonstrated that PUS1 acts as a key mediator in RCC cell biological actions. Considering the RNA-sequencing data showing the downstream cell pathways and genes that are possibly regulated by PUS1, future work is in need to elucidate the molecular mechanisms underlying PUS1 facilitating RCC progression and to figure out the possible function of PUS1 in RCC tumorigenesis in clinic.

Studies on the family of *PUS* gene in cancer are quite few. Despite previous publications showing that PUS7 was correlated with glioblastoma [31], colorectal cancer [34] and ovarian cancer [35], a recent study by Fang et al. reported that PUS1 was overexpressed in breast tumors compared with paired normal tissues, and PUS1 expression was positively correlated with triple-negative breast cancer status and tumor grade [16]. Consistent with their results showing that silencing of PUS1 significantly suppressed breast tumor proliferation and invasion, our data further made a complement for PUS1 function in tumor, providing with evidence that PUS1 would be a potential diagnose marker for cancers, including RCC. Owing to the rapid growth of our better understanding on epitranscriptomes in physiology and diseases, researches on therapeutic strategies targeting the epitranscriptomic machineries are evolving [36]. Thus, designing and developing novel inhibitors for pseudouridine synthases such as PUS1 may contribute to pseudouridine-targeting therapeutic development.

In conclusion, our findings showing the potential role of PUS1 in RCC cells provide with evidence that PUS1 is involved in RCC progression, which may help contribute to RCC diagnosis and intervention in clinic.

## Supplementary Materials

Supplementary Figure 1
