# Supplementary material for: PUS1 is a novel biomarker for evaluating malignancy of human renal cell carcinoma
Source: Aging (Albany NY). 2023 Jun 13;15(11):5215–27. doi: 10.18632/aging.204799 (PMC10292901; doi:10.18632/aging.204799)
Supplement: Supplementary Figure 1 [file aging-15-204799-s001.pdf]

SUPPLEMENTARY FIGURE

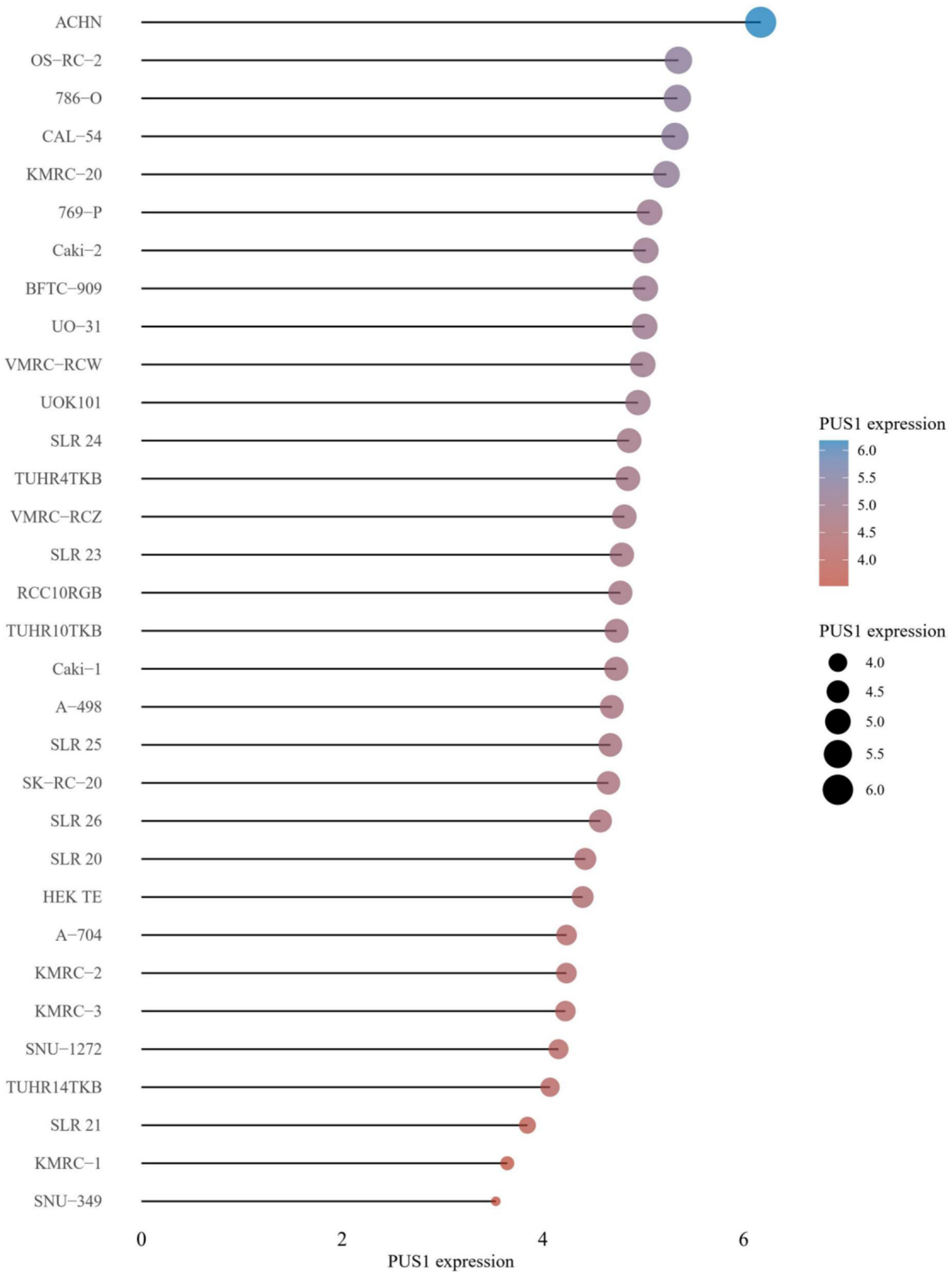

Supplementary Figure 1. Data of *PUS1* mRNA expression analysis in 33 renal cancer cell lines from the CCLE portal.
